# Time indicators of pre-hospital emergency missions in Qazvin province in 2021-2022

**DOI:** 10.5249/jivr.v16i1.1844

**Published:** 2024-01

**Authors:** Najmeh Chegini, Sajad Noorian, Mojtaba Senmar, Soheil Soltani, Mostafa Amiri, Fatemeh Rashvand, Mohadese Aliakbari

**Affiliations:** ^*a*^Student Research Committee, Social Determinants of Health Research Center, Research Institute for Prevention of Non–Communicable Diseases, Qazvin University of Medical Sciences, Qazvin, Iran.; ^*b*^ Department of Statistics, Faculty of Science, University of Qom, Qom, Iran.; ^*c*^ Emergency Medical Service Center, Qazvin University of Medical Sciences, Qazvin, Iran.

**Keywords:** Emergency, Medical Services, Pre-hospital, Time, Time Management

## Abstract

**Background:**

Pre-hospital emergency has a crucial role in providing timely care for patients. In this system, seconds and minutes mean the difference between life and death. Considering the importance of the role of pre-hospital emergency in providing services to different patients and the necessity of continuous evaluation of this system, the present study was conducted to investigate time indicators in pre-hospital emergency missions.

**Methods:**

This cross-sectional study was conducted in 2022 in Qazvin province, Iran. The research popula-tion was all the calls made to pre-hospital emergency bases in Qazvin province The required information, including time indicators and demographic characteristics of the patient, was obtained using the electronic registration system (Asayar). Data were Analysis using descrip-tive statistics and SPSS 20 software.

**Results:**

Out of the 35,943 patients admitted to the hospital, 20,915 were male while the remaining were female. The mean age of the patients was 44.09 ± 21.82 years. Accidents (29.41%) were the most common reason for contacting the pre-hospital emergency. In all transfer missions, the mean delay time (0:02:23 ± 0:03:33), response time (0:15:02 ± 0:09:42), the time on the scene (0:18:33 ± 0:11:10), total run time (0:54:02 ± 0:25:20), transport time (0:20:25 ± 0:16:49), round trip time (1:32:43 ± 1:08: 43).

**Conclusions:**

The findings of the present study provided valuable information about the variety and number of missions in a pre-hospital emergency. The results showed that some indicators are within the standard range and some indicators are far from other regions of the country and the world. Increasing the number of bases, increasing the number of ambulances, and implementation of continuous training courses for personnel can improve time indicators and increase the quality of service to different types of patients.

## Introduction

The emergency medical service (EMS) system manages the care provided to patients in pre-hospital settings.^1^ Pre-hospital emergency is essential to save the lives of patients suffering from life-threatening accidents and diseases,^2^ and it plays a crucial role in providing timely care.^3^ Pre-hospital emergency care is provided in diverse and often uncontrolled conditions, making it the front line of health services.^4^ Due to the lack of medical facilities in villages and remote areas, the demand for pre-hospital emergency services is increasing with the advancement of technology and people's health awareness.^5,6^ Prehospital indicators, including time, are essential for emergency services.^7^

By monitoring time indicators, healthcare providers can identify strengths and weaknesses to improve clinical care for the injured.^7^ Seconds and minutes can make a life or death difference in this system and can determine whether a patient will continue living a normal life or suffer from a serious disability.^8^ In addition, the success of the pre-hospital emergency care system relies on the response time and it has a vital position in the survival of patients, so one of the key concepts in providing emergency care to the injured is the golden hour after the injury, which aims to revive, support and stabilization of patients.^9^ Therefore, timely pre-hospital care is essential to prevent patient fatalities either at the scene or during transfer to the hospital.^10^ Moradian et al.'s study among stroke patients showed that time indicators are in good condition.^11^ However, Alipour and Nasiri's study showed that all the time indicators in the pre-hospital emergency, except for the time at the scene, are more than the standard limit.^12^ Jafari et al.'s study also showed that in 54 of the missions, the response time exceeds the standard.^13^

Various methods and models are employed to assess the efficiency of pre-hospital emergency care.^14^ To ensure sufficient healthcare services in both urban and rural areas, it is crucial to monitor the usage of pre-hospital emergency services in response to changing patterns.^15^ But it should be said that in a system that plays an essential role in improving the results of various time-dependent diseases,^1^ time indicators are more important than other indicators.^7^ The pre-hospital mortality rate, which can be decreased with the timely arrival of emergency services, has been reported to be as high as 40%.^7^

In general, each country should assess its EMS system and make achievable incremental improvements.^16^ Despite the great importance of pre-hospital emergencies, few studies have been done in this field. In the pre-hospital emergency of Qazvin province, pieces of evidence of different mission times are not available. However, the facilities of these systems vary between countries and regions, making time indicators particularly crucial. This study investigates pre-hospital emergency time indicators in Qazvin province.

## Methods 

**EMS in Qazvin**

In Qazvin, in case of a medical emergency, people call 115 and speak with an Emergency Medical Dispatcher (EMD) who takes the patient's medical history and address. The EMD sends the diagnosis and address to the control unit. The control unit sends mission information to the nearest base to the scene (telephone missions). There are two models in non-telephone missions. In the first model, the patient visits the base for examination. In the second model, the patient's family requests personnel to come to the site or bedside. According to the latest national census, the population of Qazvin province is 1,273,761 individuals. This province in the northwest of the country has an area of 15805 km2 and has allocated one percent of the country's area. 

This crosssectional study was conducted in 2021 (March) – 2022 (March) in Qazvin province, Iran. The research population was all the calls made to pre-hospital emergency bases in Qazvin province. All missions where the patient was transferred to the hospital and admitted were included in the study. If the information in the Asayar software (a software approved by the Ministry of Health of Iran, which is used for electronic registration of EMS missions throughout Iran) is incomplete, that mission will be excluded from the study.

**Data collection tools**

Data was collected using Asayar software. The in-formation in this software included two parts. In the first part, patients’ information, the reason for the call, the technicians’ diagnosis, and location type are recorded. The second part includes pre-hospital emergency time indicators such as delay time, response time, on-scene time, total run time, transport time, and round trip time.^10,11^ ASAYAR Automation System is responsible for managing and controlling the service delivery process in the country's emergency organization from the moment the client calls to the end of the mission. This product has been designed, produced, and used for the first time in the country based on the needs of Iran's Emergency Organization. According to the instructions of operational processes of pre-hospital emergencies in Iran, the standard duration of response time index is 14 minutes in suburban missions, 12 minutes in metropolitan cities, and 8 minutes in other cities. This standard is less than 20 minutes for on-scene time.

**Analysis**

The data were analyzed using descriptive (Mean, Std. Deviation, and Percent) statistics and SPSS software (version 25).

## Results

Out of the 35,943 patients admitted to the hospital, 20,915 were male while the remaining were female. The mean age of the patients was 44.09 ± 21.82 years. The triage expert diagnosed the most common causes of contact as follows: accidents (10571 cases), weakness and lethargy (3476 cases), and cardiovascular (3899 cases) (Table 1). 22442 missions were urban and the rest were road missions. Most of the patients were transferred to Rajaei Hospital, the province's trauma center, and the rest of the patients were transferred to other treatment centers based on the type of problem. In all transfer missions, the mean delay time (0:02:23 ± 0:03:33), response time (0:15:02 ± 0:09:42), the time on the scene (0:18:33 ± 0:11:10), total run time (0:54:02 ± 0:25:20), transport time (0:20:25 ± 0:16:49), round trip time (1:32:43 ± 1:08: 43). In road and urban missions, the response time is (0:17:34 ± 0:12:26) and (0:13:31 ± 0:07:11) respectively (Table 2 ). This index is (0:12:57 ± 0:11:04) and (0:15:11 ± 0:09:35) in non-telephone and telephone missions, respectively (Table 3 ).

**Table 1:** Frequency and percentage of missions.

Diagnosis	Frequency	%	Diagnosis	Frequency	%
Accident	10571	29.41	Fire and explosion	98	0.27
Weakness and lethargy	3476	9.67	Cancer	12	0.03
Respiratory	3187	8.86	Drowning in water	7	0.01
Loss of consciousness	2218	6.17	Being locked up in a closed place	2	0.005
Trauma	2565	7.13	Electrocution	76	0.21
Cardiovascular	3899	10.84	Animal bites and stings	120	0.33
Falling from a height	1355	3.76	Abdominal pain	1068	2.97
Convulsions	965	2.68	Stroke	652	1.81
Conflict and fighting	1510	4.20	Mental	398	1.10
Dizziness	349	0.97	Poisoning	922	2.56
Diabetes	262	0.72	Burn	156	0.43
Hemorrhage	403	1.12	Pain	344	0.95
Obstetrics and Gynecology	263	0.73	Allergy	78	0.21
Probably dead	120	0.33	Other	867	2.41
	Total			35943	

**Table 2:** Mean and standard deviation of time indicators in urban and road missions.

Type of mission		Delay Time	Response Time	Scene Time	Transport Time	Round Trip Time	Total Run Time
Urban	**N**	22442	22442	22442	22442	22442	22442
	**Mean**	0:02:15	0:13:31	0:18:05	0:15:14	1:17:28	0:46:50
	**Std. Deviation**	0:03:03	0:07:11	0:10:32	0:10:06	0:48:49	0:17:20
Road	**N**	13501	13501	13501	13501	13501	13501
	**Mean**	0:02:38	0:17:34	0:19:21	0:29:03	1:58:04	1:05:59
	**Std. Deviation**	0:04:15	0:12:26	0:12:05	0:21:32	1:27:06	0:31:18

**Table 3:** Mean and standard deviation of time indicators in non-telephone and telephone missions.

Type of missions		Delay Time	Response Time	Scene Time	Transport Time	Round Trip Time	Total Run Time
Telephone	**N**	33633	33633	33633	33633	33633	33633
	**Mean**	0:02:26	0:15:11	0:18:35	0:20:16	1:32:15	0:54:03
	**Std. Deviation**	0:03:35	0:09:35	0:10:55	0:16:41	1:08:28	0:25:04
Non-Telephone	**N**	2310	2310	2310	2310	2310	2310
	**Mean**	0:01:44	0:12:57	0:18:06	0:22:37	1:39:39	0:53:41
	**Std. Deviation**	0:03:07	0:11:04	0:14:12	0:18:23	1:12:03	0:28:54

## Discussion

The present study, which was conducted to investigate the time indicators of Qazvin pre-hospital emergency missions, showed that the average response time in all missions is more than the standard. However, this index is lower for urban missions and higher for road missions than the standard range. A systematic review study showed that the mean response time is 7.3 minutes in Asia, 8 minutes in Oceania, 19 minutes in Africa, and 9 and 11 minutes in America and Europe.^17^ In other words, the response time in most continents is lower than the mean response time in the present study. These differences can be attributed to the type of study above because the systematic review has considered the mean of this index in different countries. The articles of this systematic review^17^ in the countries of Brazil, the United States, Spain, and Poland show that the response time index is higher than in Qazvin province. In Jafari et al.'s study,^13^ the mean response time was 9.01 minutes, while in the study of Jarvis et al.^18^ the response time for trauma patients during the COVID-19 pandemic was 7 minutes. In other words, the response time in the two above studies is less than the response time of the present study. It seems that considering a certain group of patients has led to the difference between the results of the above studies and the present study.

In the study of Zeraatchi et al.^19^ which reviewed the files of 742 patients, the mean delay time was 0.02 minutes. In other words, the results of the two studies are somewhat consistent. In Yadollhi's study,^10^ the mean delay time was 0.01 minutes, which is less than the mean delay time in the present study. It seems that the sample size and the use of an electronic registration system are the reasons for the difference in the results. Yadollahi's study^10^ was conducted before the implementation of the electronic registration system (ASAYAR), which is considered more accurate than paper registration. Asadi et al.^7^ reported a mean delay time of 1.01 minutes. The above study examined the missions of 12 pre-hospital emergency bases in Ardabil city, while the present study examined all the bases in the province. 

The time of being on the scene in the present study was less than the national standard. In Australia,^20^ the mean on-scene time was 34 minutes, which is longer than in the present study. Various factors such as the severity of the patient’s condition, the need for intubation, and trauma missions may prolong the time spent on scene.^21^ Attributing the time difference to the skill and speed of the personnel may be far from mind, but since the current study surveyed all types of missions, it can be concluded that personnel in this study spend less time at the scene and make decisions more quickly. Therefore, in this time index, they have better results than Fok et al.'s study. In Ashburn et al.'s study,^22^ the mean on-scene time was 14.2 minutes. Although the difference between the results of the two studies is less than 5 minutes, it should be said that Ashburn and colleagues only considered trauma patients. Apart from this, it should be said that the care and treatment needs of patients and the speed of personnel's performance are important factors affecting this time index. Almost in line with the present study, Jarvis et al. reported the duration of being on the scene during the Covid-19 pandemic in trauma patients as 16 minutes .^18^

Although there is no national standard for transfer time, it is recommended that the patient be transferred to the hospital as soon as possible. The transfer time in the study by Asadi et al.^7^ (mean: 12.53 minutes) is lower than the mean of this time index in the study of researchers. It should be taken into account that Asadi et al. have examined the missions of the base of Ardabil city. Of course, the base of the city is closer to the medical centers and has reduced the transfer time in the above study. In Ashburn et al.'s study,^22^ the mean transfer time was reported as 17.5 minutes, which is less than the transfer time in the present study. In another study in America,^18^ the duration of transferring trauma patients to a level-one trauma center during the COVID-19 pandemic was reported 17 minutes. It seems that the transfer of patients to specific trauma centers has led to a reduction in the transfer time of patients. In both mentioned studies, trauma patients were transferred to trauma centers with certain levels. The transfer of trauma patients to trauma centers is done to reduce pre-hospital time and increase the survival rate.^9^ However, in the world, the level of trauma patient care during transfer in the emergency is very different and has been discussed.^9^ In addition, the condition of the roads, driving speed, and proximity to medical centers are effective factors when transporting patients. Therefore, it can be said that the mentioned cases are important factors in the difference in the results of different studies.

Contrary to the findings of our study, In the pre-hospital emergency of Ardabil,^7^ the total run time was 15.35 minutes, which is 18.87 minutes less than the results of our study. Although this difference is significant, it should be considered that the above study only examined the missions of one city. In another study, the mean total run time in 742 transferred patients was reported to be 20 minutes,^19^ which is very different from our study. The above study used a paper registration system and in the present study, an electronic registration system was used. One of the causes of this difference can be attributed to the increase in accuracy and precision of the electronic registration method. In a study in America,^18^ the interval between contacting dispatch and reaching the hospital was reported to be 43 minutes, which is somewhat in line with the findings of our study.

In the pre-hospital emergency of Zanjan,^19^ the total time of the mission was reported to be 94 minutes, which is nearly coincidental with the results of the current study, which is 92.43 minutes. In another study, the duration of this index is 50.52 minutes,^7^ which is a remarkable difference from the findings of our study. The total time of the mission in the study of Yadollahi et al.^10^ is also less than the mean of this time index in the present study. According to the research done in the method section of the above studies, it should be said that the total time of the mission is influenced by other times. In other words, it should be said that the shorter the distance between the pre-hospital emergency base and the scene and the scene to the hospital, the shorter the entire mission time. Since all regions of the province have been investigated in the current study, it seems that the increase in the total mission time can be justified. Apart from this, it should be considered that the current study was conducted during the COVID-19 pandemic. Personal protective clothing and patient screening during the COVID-19 pandemic can increase times.^18^

The findings showed that all-time indicators in urban missions are less than in road missions. Although the speed of service delivery is important in preventing death and disability, the number of bases, the type and number of ambulances, the wrong location, and the distance from the patient's bed are factors that cause delays and obstacles related to distances.^13,23^ It seems that urban areas have had a proper distribution of these obstacles and restrictions, and this has led to the reduction of time indicators in urban missions. In addition, there are more urban bases in Qazvin. And in many urban missions, the distance between the patient and the hospital is closer. This could have led to the reduction of time indicators in urban missions.

In our study, the mean of delay time, response time, scene time, and total run time are higher in telephone missions than in non-telephone missions. In other words, when the patient's family personally goes to the pre-hospital emergency base, the indicated time indicators become shorter. Telecommunications problems, including many radios and network black spots, as well as a large number of emergency numbers, are other important factors that confuse the general public when calling EMS. These telecommunication problems also affect EMS personnel and create another significant barrier to rapid notification.^23^ Therefore, it must be said that when the patient or the patient's companion visits the pre-hospital emergency base in person, many of these obstacles are removed, and this leads to a decrease in these time indicators in non-telephone missions compared to telephone missions. In telephone missions, the mission must be announced to the base using telecommunication and internet systems, while these systems play a lesser role in non-telephone visits.

**Limitations**

One of the main limitations of the study is not considering interventions and care that can affect time indicators. Another limitation of the study was the lack of a specific standard for all time indicators, which made interpretation and analysis of data difficult. Although some indicators, such as response time, have a national standard. However, it seems that the exact determination of the time standard requires consideration of various issues such as the number of ambulances, the number of bases, and the number of personnel.

**Strengths**

The most important strength of the present study is the size of the studied sample, which increases the validity and reliability of the results.

## Conclusion

EMS personnel are at the forefront of treating and caring for patients in different parts of the country. The findings of our study apportioned helpful information about the status of time indicators and the variety and number of missions in pre-hospital emergencies. The results showed that some indicators are within the standard range and some indicators are far from other regions of the country and the world. Policymakers and managers are expected to improve time indicators in a pre-hospital emergency care system by updating equipment and facilities and increasing the number of ambulances and bases. It is suggested that the role of interventions and care performed by pre-hospital emergency personnel in these indicators should be considered in upcoming studies. According to the sample size of this study, the results can be utilized in similar contexts and environments.
